# Current concepts in robotic total hip arthroplasty

**DOI:** 10.1051/sicotj/2020041

**Published:** 2020-11-27

**Authors:** Pascal Kouyoumdjian, Jad Mansour, Chahine Assi, Jacques Caton, Sebastien Lustig, Remy Coulomb

**Affiliations:** 1 Centre Hospitalo-universitaire de Nîmes Rue du Pr. Robert Debré 30029 Nîmes France; 2 Université Montpellier 1 2 Rue de l’École de Médecine 34090 Montpellier France; 3 Laboratoire de Mécanique et Génie Civile (LMGC), CNRS-UM1 860 Rue de St-Priest 34090 Montpellier France; 4 Department of Orthopedic Surgery, Lebanese American University-Rizk Hospital Beirut Lebanon; 5 Institut de chirurgie orthopédique Lyon France; 6 Centre Albert-Trillat, CHU Lyon Croix-Rousse, Hospices Civils de Lyon 69004 Lyon France

**Keywords:** Robot, Navigation, Surgery, Hip, Total Hip Replacement, THA, Planning

## Abstract

*Introduction:* Total hip replacement provides mostly fair functional and clinical results. Many factors play an essential role in hip stability and long-term outcomes. Surgical positioning remains fundamental for obtaining accurate implant fit and prevent hip dislocation or impingement. Different categories of robotic assistance have been established throughout the previous years and all of the technologies target accuracy and reliability to reduce complications, and enhance clinical outcomes. *Materials and methods*: An overview is proposed over the principles of robotic assistance in hip arthroplasty surgery. Accuracy, reliability, management of the bone stock, clinical outcomes, constraints and limits of this technology are reported, based on recent literature. *Results*: Potential advantages regarding pre-operative planning accuracy, cup positioning, maintenance of the center of rotation, preservation of an adequate bone stock nay clinical short- and mid-term outcomes are balanced with some reported disadvantages and limits like hip anatomical specificity, cost-effectiveness, engineering dependence. *Discussion*: The use of robotic-assisted THA presents clear and evident benefits related to accurate implant positioning and maintenance of a minimal bone while allowing. For some authors, an early improvement in functional results and patient’s recovery. This technology demonstrated a shorter surgical time and a short learning curve required to optimize its use and this technology presents promising outcomes and results and potential use in routine clinical application but its limitation of use is still present especially the cost of the robot, the need for the presence of an engineer during the surgery, its availability of use in all hospitals as well as the difficulty presented in dysplastic or dysmorphic hip joints.

## Introduction

Total hip replacement remains the main arthroplasty modality that provides greatest functional and clinical outcomes. However, patient’s lifestyles and daily requirements are constantly evolving. Short- and long-term outcomes do not always meet their expectations. Associated complications or poor results may prevail.

A number of factors including patient’s personal data, primary surgeries, surgical techniques, and types of implants may influence the course of this surgery. Some of these factors remain related to the surgeon’s preoperative planning as well as his ability to reproduce his pre-operative templates during surgery [1].

Many factors play an essential role in hip stability, implant fit, and osseointegration such as the material’s properties, the interface’s porosity, and the implant’s geometry [2–4].

The implants insertion appears to be one of the fundamental factors for implant survival. Poor positioning was correlated to a higher rate of intra-prosthetic or periprosthetic dislocation [5]. In addition, impingement, pain, leg length discrepancy, accelerated implant wear, loosening [6], and finally poor functional outcomes, and increased surgical revision rates were also correlated to poor implant positioning. They make up to 40% of the causes of revision surgery [7]. However, the manual procedure is reported as highly dependent on:

- The large variations in the patient’s initial pelvic positions on the operating room table and significant pelvic movement during the intraoperative range of motion testing [8].
- Surgical expertise [9] with 38%–47% of acetabular implants not being placed according to the surgeon’s choice [10, 11].

Pre-operative planning or use of hip navigation partially optimizes the placement of the cup [12–14] without significant difference between computed tomography (CT)-based or imageless navigation [15]. Some authors reported no improvement while using the navigation on cup implantation compared to the freehand technique of positioning [16]. However, these instruments do not provide full control of the planned position of the implants. This could lead to a decreased accuracy of positioning during acetabular reaming, cup impaction, and femoral stem implantation by the surgeon’s hand motions. A recent Medicare database analysis failed to demonstrate any clinically significant reduction in the short-term adverse events with the use of computer-assisted surgery [13].

Analysis of implant positioning with conventional surgeries that do not use intraoperative navigation system reported a consistent modification of the cup center of rotation (COR) compared to the native acetabulum. The choice of the acetabular floor as the anatomic landmark during the reaming can modify the COR medially and superiorly making it difficult to compensate by using a high offset stem [17]. The ideal depth of acetabular reaming is mostly based on the lateral border of the teardrop and dependent on its thickness [18]. However, free hand techniques do not secure and allow adapted control of the depth reaming which is mostly dependent on bone quality. If navigation allows better control of the cup compared to a free hand technique, postoperative CT imaging evaluation of Navigation data using the Lewinnek plan during assisted procedures confirmed the same trend [19].

In recent years, robotic technology has been developed in different manufacturing areas. This technology has recently reached the medical field. Robotics is now being used in different orthopedic subspecialties; particularly in knee, spine, and hip surgeries [20]. This type of assistance allows more direct and consistent control of the acetabular reaming and positioning of implants during the surgical procedure. Targeted goals are reduction of outliers of the different defined safe zone position [5, 9, 21] and improvement of clinical outcomes. This overview includes the principles of robotic assistance in hip arthroplasty surgery, its evolution, and actual results concerning its potential advantages and disadvantages reported in the literature.

### Types of Robots and applications in hip replacement

The robot is a mechatronic device (mechanical, electronic, and computer) designed to perform a variety of tasks that have been programmed in advance. This definition mainly highlights the role of cup and stem position planning. The static nature of skeletal anatomy simplifies preoperative imaging, hence the advantage of the use of this type of technology.

Different categories of robotic assistance have been developed throughout the previous years [22] with different principles, mechanisms of action, and bone cutting methods (Table 1).

**Table 1:** Evaluation of cup position in comparative studies of the literature between with and without robotic-assisted system.

	Date	Type of study	Country	Number of procedures		Robot type	Population gender			Surgical approach			Age (y)			Number of complications			FU	Outcomes studied
				No Robotic	Robotic		Non Robotic		Robotic	Non Robotic		Robotic	Conventional		Robotic	Non Robotic		Robotic		
Lim et al [46]	2015	RCT	Korea	25	24	ROBODOC	M 13 F 12		M 11 F 13	NR		NR	45.6 (21–65)		51.2 (19–67)	2 (intraPPF)		0		Functional scores, complications, stem alignment, LLD, operation time
Siebel and Kafer [75]	2005	Retrospective	Germany	35	36	CASPAR	M 19 F 16		M 21 F 15	Lateral		Lateral	60.6 ± 7.0		58.9 ± 8.9	3 (1 dislocation + 2 intraPPF)		4 (2 dislocation + 1 neuro +1 infection)	18	Functional scores, complications, operation time
Nakamura et al [43, 82]	2010	RCT	Japan	71	75	ROBODOC	M 10 F 51		M 13 F 56	Post.		Post.	58 ± 9		57 ± 10	10 (1 dislocation + 4 thigh pain + 5 intraPPF		7 (4 dislocation + 3 thigh pain)	min > 4 Y	Complications, LLD, operation time
Domb et al [83]	2014	Retrospective matched-pair controlled	USA	62	69	MAKO	M 19 F 31		M 19 F 31	Post.		Ant. (fDAA)	56.7 ± 8.1		56.8 ± 7.9	0		1 (intracup malposition)	–	Complications, cup angle, safe zone of cup, operation time
El Bitar et al. ** [84]	2015	Retrospective	USA	88* (2 groups)	67	MAKO	M 12	M 23	M 29 F 38	fDDA	Post.	Posterior 67	58 ± 12.3	55.3±9.3	60.2 ± 9.6	NR		NR	–	Leg length discrepancy
							F 17	F 36		29	59									
Tsai et al. [24]	2016	Retrospective	USA	14	12	MAKO	M 7 F 7		M 2 F 10	Post.		Posterior	58.7 ± 7.5		61.4 ± 8.9	0		0	NR	Cup angle, safe zone of cup, stem alignment
Bargar et al. [41]	2018	RCT	USA	22	45	ROBODOC	M 12 F 10		35 M 10 F	Post.		Posterior	59.8 ± 9.4		59.1 ± 8.2	0		0	14 Y	Functional scores
Schulz et al. [44]	2007	Retrospective	Germany	–	128	ROBODOC	–		NR	–		Lateral	–		56 (19–75)	–		17 intra op (9 technical, 8 other) 9 postop	3.8 Y	Complication, Clinical outcomes
Suarez-Ahedo et al. [57]	2017	Control study	USA	57	57	MAKO	M 20 F 37		M 20 F 37	Ant. & Post.		NR	56.9 (38.8–72.3)		56.9 (40.6–73.4)	NR		NR		Bone preservation
Domb et al. ** [38]	2015	Retrospective	USA	1752* (multi groups)	228	MAKO	–		–	DAA and post. +/− Xray+/− Nav		Ant. (DAA) & Post.	64.7 ± 11.9		58.6 ± 10.8	NR		NR	–	Cup angle, safe zone of cup, leg length discrepancy
Bargar et al. [45]	1998	Retrospective	Germany	62	65	ROBODOC	NR		NR	Post.		Post.	NR		NR	9 (4 dislocation +3 intraPPF, +2 other)		9 (4 dislocation + 2 neuro + 1 loosening +2 other)	1 at 2 Y	Complication
Hananouchi et al. [85]	2007	Retrospective	Japan	27	31	ROBODOC	NR		NR	NR		NR	57.4 ± 7.1		56.7 ± 9.2	NR		NR	24 M	Functional score, complications, stem alignment
Honl et al. [42]	2003	Randomized controlled	Germany	80	61	ROBODOC	M 24 F 56		M 24 F 37	Anterolat.		Anterolat	70.7 ± 8.3		71.5 ± 7.1	6 (3 dislocation + 1 neuro + 2 infection)		15 (11 dislocation + 4 neuro + 1 HOssif.)	2 Y	Functional score, complications, stem alignment, operation time
Kamara et al. ** [51]	2017	Retrospective	USA	296* (2 groups)	98	MAKO	M 93	M 43	M 45 F 53	M.Post	DAA	Post.	NR		NR	7^a^	5^b^	2^c^	NR	Complications, cup angle, operation time,
							F 105	F 55												
Nishihara et al. [40]	2006	Retrospective	Japan	78	78	ORTHODOC	M 14 F 64		M 14 F 64	Post.		Post.	58 (29–77)		58 (27–81)	5 (PPF)		0	2.3 Y	Functional score, complications, stem alignment, operation time
Heng et al. [73]	2018	Retrospective	Australia	45	45	MAKO	M 32 F 13		M 25 F 20	Post.		Post.	62.8 ± 12.3		64.5 ± 9.9	3 (acetabular fractures)		2 (1 conversion +1 wound infection)	–	Intraoperative complication, operation time bleeding LOS
Kanawabe et al. [54]	2015	Prospective	USA	–	43 (38)	MAKO	–		M19 F 24	–		Post.	–		63 (48–79)	–		0 (5 technic failures)	–	Intraoperative complication, cup position accuracy
Kayani et al. [72]	2019	Prospective	UK	50	25	MAKO	M 26 F 24		M 13 F 12	Post.		Post.	69.4 ± 5.2		67.5 ± 5.8	0		0	6 W	cup angle accuracy, early complication (6weeks)
Kong et al. [52]	2020	Retrospective	China	100	86	MAKO	M 40 F 60		M 36 F 50	Post.		Post.	51.9 ± 12.6		51.9 ± 10.8	NR		NR	–	Learning curve, cup angle
Banchetti et al. [86]	2018	Retrospective	Italy	51/100***	56/120***	MAKO	M 26 F 25		M 31 F 35	Post.		Post.	69.8 ± 10.2		66.2 ± 11.1	NR		NR	2 Y	Complications, PROMS, clinical outcomes
Perets et al[77]	2017	Retrospective	USA	–	162	MAKO	–		M 73 F 89	–		Ant. & Post.	–		61.2 ± 8.9	–		12 (6 intraPPF + 1 infection+ 1 neuro+4 other)	>2 Y	PROMS, clinical outcomes
Illgen et al. ** [49]	2017	Retrospective	USA	200* (2 groups)	100	MAKO	M 42	M 50	M 42 F 58	Post.		Post.	65 ± 14	60 ± 12	62 ± 11	Dislocation		Dislocation: 0	2 Y	Cup position, operative time, blood loss, complication
							F 58	F 50		Early THA	Late THA					5	3			

* Sum of all procedures without the use of the robot in studies with more than two groups of comparison including 2 techniques assistance or approach of non-robotic THA.

** Relating studies with multi-subgroups comparing different THA procedures without robot with a group with a robotic assistance.

*** Respondent’s patient at the follow-up.

a 3 intraoperative (femoral fractures) + 1 early dislocation + 4 revisions (1 femoral for the failure of osseointegration, 1 LLD, 1 wound infection, 1 delayed infection at 1 Y;

b no intraoperative, 2 anterior recurrent dislocation, 3 revisions (1 psoas tenotomy, 1 metallosis with CoC, 1 ALVAL with modular neck),

c no intraoperative complication, 1 early recurrent dislocation with revision, 1 revision for subsidence

*Abbreviations*. NR: non reported, intraPPF: intraoperative periprosthetic fracture, neuro: neurologic complication as palsy or lesion, post.: posterior approach, ant.: anterior approach, anterolat, HOssif.: Heterotopic ossification. Antero lateral approach DAA: Direct Anterior Approach, fDAA: DAA assisted by fluoroscopy, X-ray: surgery assisted by intraoperative fluoroscopy, Nav: surgery assisted by navigation, W: week, M: month, Y: year, –: No way in the goal of the study.

Systems such as Passive systems (ex da Vinci^®^ surgical system), semi-active systems controlling and adjusting in real time the surgeon’s hand (robotic-arm assisted surgery) MAKO^®^ THA system (Stryker^®^, Mahwah, NJ) [23] or active systems (able to manage a task independently without direct human manipulation due to the use of preprogrammed algorithms and defined parameters of bone resection) have been developed. The first active robotic system used in hip replacement surgery was the ROBODOC (Think Surgical^®^) surgical system [16], robotic assistance without continuous control by the surgeon throughout the procedure. Different mechanisms of action have been developed, particularly for knee and hip surgery with the direct cutting of bone to the final planned cut or indirect planning landmarks to adjust placement or holding of cutting jigs. The robotic cutting methods can be divided into three different categories as reported by the recent literature [20]: (1) autonomous – cutting without any human hand control; (2) haptic control – cutting, milling, or drilling requiring human interaction to maneuver the robot – the robot’s movement is constrained by an end limit [24, 25]; (3) boundary control – surgeon interaction is necessary to handle the robot, but robot action is deactivated and/or the procedure is stopped if the resection exceeds the planning limits (ex Navio Surgical System^®^, Smith & Nephew^®^), but this system does not constrain the surgeon’s hand [26, 27].

### Planning: use of 3D imaging

Planning remains the key point in the use of robotic assistance. All past and current systems labeled as surgical robot require 3D imaging by a calibrated preoperative CT scan. It allows specific reconstruction of the native anatomy and adapts in a personalized way the surgical processes. Preoperative planning using 3D imaging data avoids the error of the 2D planning as vagueness magnification of the acetabulum or the femoral canal shape. Specific prior information is collected via a pre-operative CT scan acquisition, calibrated in terms of field (inclusion of knees in the field of exploration for evaluation of the femoral version). A 3D virtual model of the pelvis and femur incorporating anatomical data and the patient’s specific pelvic reference points is the base for preoperative planning [28].

The software of the robotic arm offers the ability to plan the position of implants using the patient’s anatomical landmarks with respect to the native acetabular geometry, the acetabular center of rotation, the femoral version, the center of rotation induced by the planned stem, the combined offset of both and the comparative leg length of the operated hip.

The primary key point prior to the procedure is the surgeon’s planning confirmation. Dedicated engineers are present during the intervention to assist the surgeon. They are able to perform any changes in the design pre- or intraoperatively. The data measured are:

- The acetabular component orientation.
- The center of rotation.
- The cup overflow surface (especially anterior).
- Planning a cemented or cementless, anatomical or straight, reversed or neutral pivot stem.
- Combined lateralization (offset).
- Assessment of combined anteversion considering current data on safe zones.
- Bone preservation in decreasing the milling diameter and choosing a smaller sized cup while respecting the previous items.
- Assessment of the lower limbs length.

### Robot for THA (Figure 1)

Three main robotic systems have been used in hip replacement surgery. The first one was an automated system (ROBODOC) developed in 1992 [4]. ROBODOC was the first active robot performing surgical procedure without direct surgeon guidance. Used in the United States during 1994–1995 with FDA approval and for clinical practice in Germany since 1994, the goals were to improve the choice of acetabular cup size and the accuracy of its implantation as well as to optimize the femoral cavity preparation in order to limit the risk of periprosthetic fracture and ensure a better stem alignment. ROBODOC consisted of a preoperative planning computer workstation ORTHODOC (Integrated Surgical Systems, Davis, CA, USA), provided with a robotic arm containing five mechanical freedom axes and armed with a high-speed milling device [29]. With a 3D preoperative planning based on CT data, the system could enable the optimal design for each patient to be selected by comparing the fit and fill with several designs [30]. Press fit and capacity for the femur to reconstruct the true anatomic situation have been reported in a fresh human cadaveric femoral study by Jerosch et al. [31].

**Figure 1:**
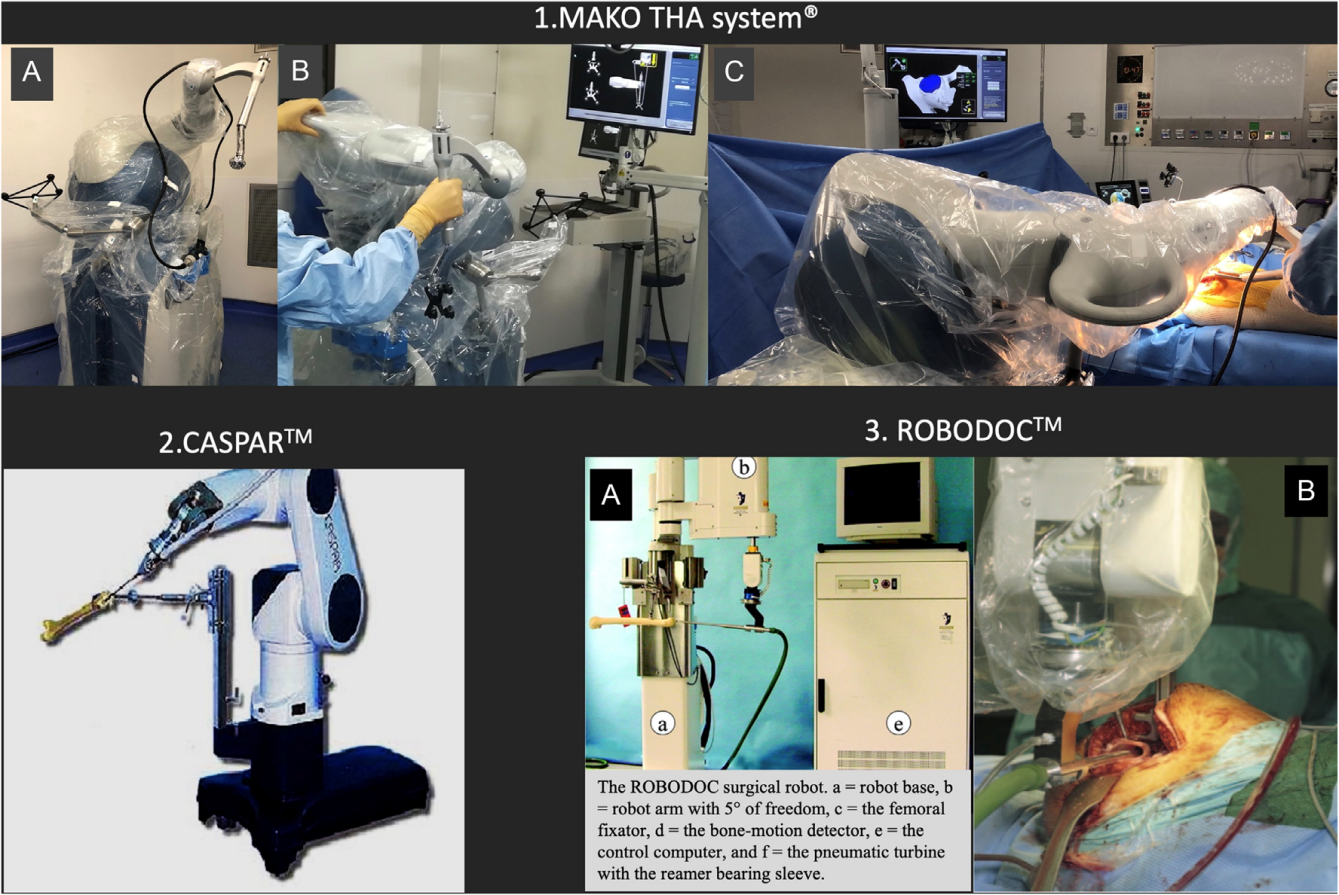
Different type of robotic-assisted system used for THA: 1. MAKO^TM^ THA system (Stryker^®^, Mahwah). (A) Description, (B) Preoperative calibration of the system, (C) During the final implantation of the cup. 2. CASPAR (OrthoMaquet^®^/URS Ortho) adapted to hip surgery. 3. ROBODOC system (ISS, THINK Surgical^®^). (A) Description [42], (B) During femoral canal milling process [81].

The procedure sequences consist of: first, the preoperative time with pin implantation, CT scan, preoperative planning, workstation surgical setup in the operating room, and then surgical time with sequential exposure, pin location, registration, and robotic milling of the femoral cavity.

Similar to ROBODOC, the CASPAR computer assistance (Universal Robot Systems; Rastatt, Germany, OrthoMaquet^®^/URS Ortho) based on a preoperative CT scan was used to automatically mill the femoral canal and fix the stem. After few studies reporting accurate femoral preparation and stem positioning [32, 33], this system is currently aborted because of its poor results and its higher rate of complications [34].

The most currently robotic assistance used actually for THA is the MAKO THA system (Stryker^®^, Mahwah, NJ). MAKO Surgical was founded in 2004 for medical applications, amongst a wide variety of other computer-assisted surgery technologies. First adapted for partial knee replacement, with the first procedure performed in 2006, the development for its use in THA was performed in October 2010. This robotic assistance received the FDA clearance in 2015 for new enhancements in THA application of its MAKO Rio surgical robot. An increase in the use of this system is now reported for THA, PKA, and TKA [20].

Also, this direct haptic system is based on a 3D CT acquisition. MAKO THA system (Stryker^®^, Mahwah, NJ) was introduced with the purpose of providing more accurate implant positioning, alignment according to plan, perfectly related to the selected pre-operative planning considering, among other things, the choice of restoration or not of the hip centre of hip rotation, bone economy, adjustment of the equality of the lower limbs, control of the overall offset, the absence of intraprosthetic conflict and this objective is fully integrated into the will to improve the functional outcome in the short-, medium- and long-term.

The analysis of a preoperative CT scan by a patient-specific computer-aided design (CAD) software modeling the pelvis and proximal femur and identifying specific anatomical landmarks, is used to accurately determine the position of these anatomical points, the elective plane of planning, and the pelvic tilt during the surgery. Acetabular orientation is closely dependent on the referential plane used. Placement of the acetabular component is based on the functional (coronal) plane defined by Murray [35]. In opposition to the functional plane, the anatomical plane does not take into account the pelvic tilt [36, 37]. Bone registration is then performed. This process enables the MAKO Total Hip application to accurately locate and track the patient’s position during surgery with respect to the placed arrays. Bone registration is a multi-step process making all CT-based models mapped to the patient’s bones.

### Surgical procedure

The total hip arthroplasty application “MAKOplasty THA^®^”; Stryker is designed to adapt to direct postero-lateral and anterior approaches, they:

- Express the use of robotic assistance for the acetabular cup and navigate the femoral stem.
- Enhance the ability to navigate the femoral osteotomy line and the femoral rotation.

**Figure 2:**
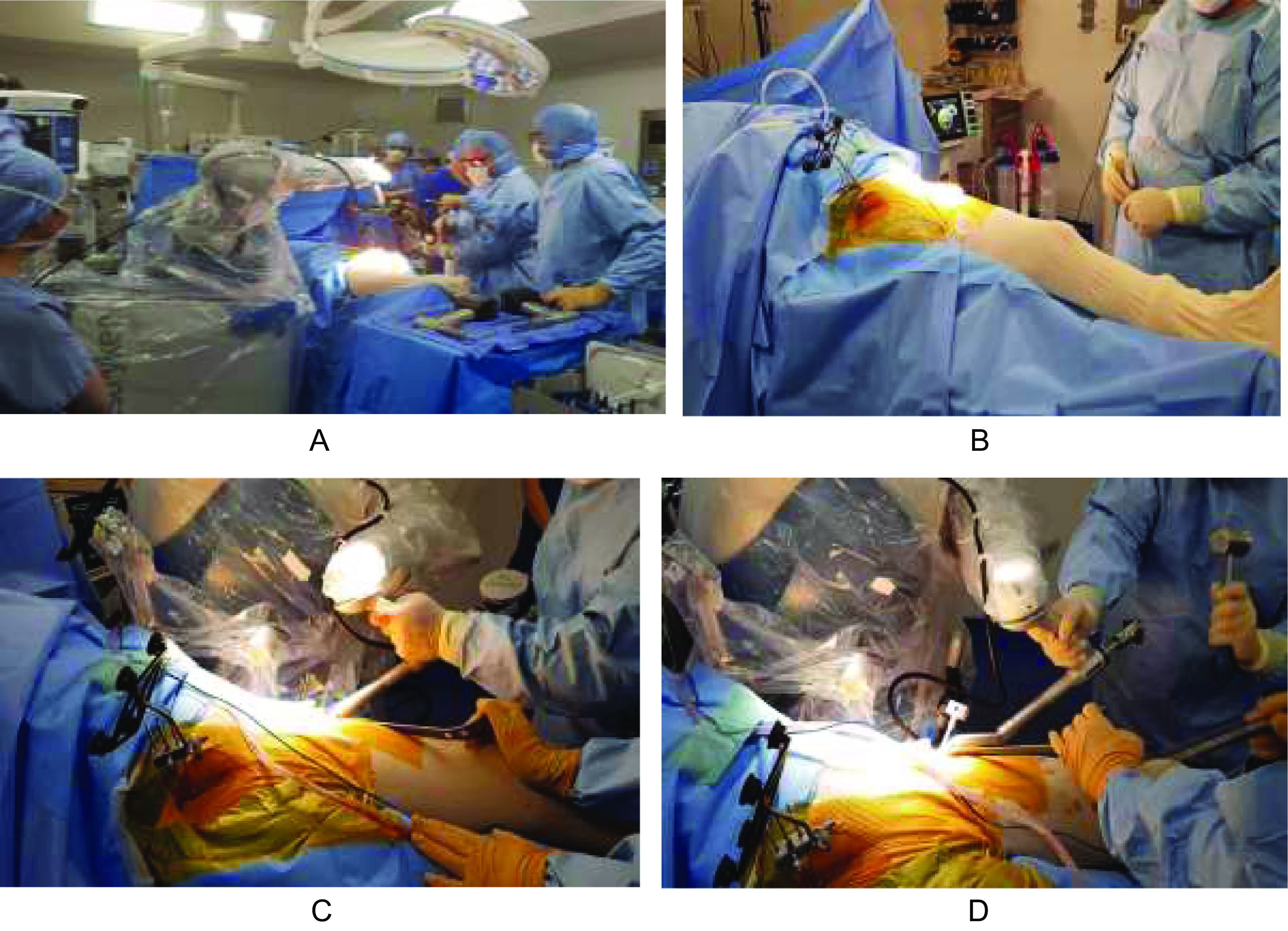
Installation of the robot (Mako^TM^ system) and landmarks during a postero-lateral approach) (A) or a DAA approach (B); during the reaming (DAA) (C) and impaction (D) process.

The surgeon remains responsible for the appropriate approach (Figure 2), type, and size of the incision for an optimized procedure. The first step is to place three pelvic threaded pins into the thickest portion of the iliac crest to hold the pelvis arrays (VIZADISK; optical trackers connected to hip trackers) and track the patient’s motion during the procedure (pins move with the pelvis).

In case of the use of posterior approach (Figure 2A), THA is performed in a lateral decubitus position. Prior to surgical prepping and draping, a distal landmark is placed on the patella slightly inferior to its center. After draping, pins are placed into the ipsilateral iliac crest. After exposure, femoral registration is performed and verified by the system.

**Figure 3:**
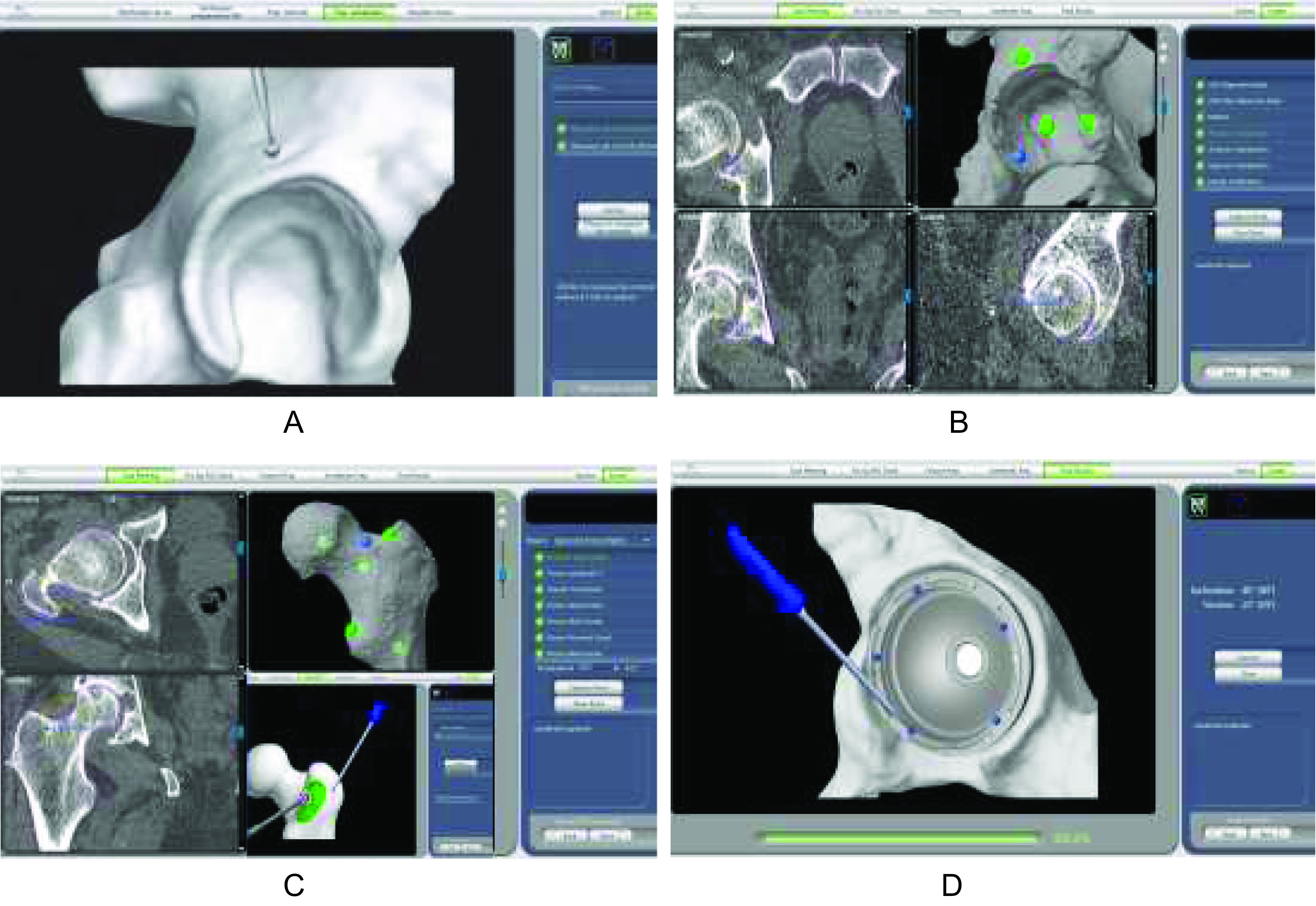
Intraoperative procedure: positioning of the acetabular landmarks (A), pre-operative mapping process of the acetabulum (B), the proximal femur (C) during an enhanced THA procedure with the positioning of the femoral marker before its mapping, control of the final acetabular cup positioning using the pointer (D).

A femoral landmark is then placed on the greater trochanter (Figure 3). Registration of the native combined offset and leg length is performed. A registration error of more than 1 mm indicates that the verification process failed and the femur must be re-registered. Once the femoral neck cut is completed. A periacetabular landmark is placed above the acetabulum postero-superiorly. Registration of the acetabulum by a mapping acquisition is merged with a calibrated pointer. The acetabulum is then reamed for cup placement using a haptic robotic arm guide acetabular reaming for cup insertion. There is a possibility of two courses.

The first course is choosing the express procedure, which allows the cup to be robot-assisted, and the size of the stem and the neck to be planned. The other is called the enhanced procedure, which also offers the ability to navigate the femoral osteotomy level and implantation of the stem by a mapping registration of the proximal part of the femur in order to optimize the adjustment of the femoral version. In this case, the femoral mapping will precede acetabulum reaming and cup insertion.

In case of the use of the direct anterior approach (DAA), THA is performed in a supine position. Some authors use a specific traction table [38]. A standard table without traction can be used (Figures 2B–2D). Three pelvic threaded pins are placed in the contralateral iliac crest. The femoral landmark is then placed in the anterior and inferior part of the greater trochanter. The procedure is carried out the same way as that described above. The enhanced procedure is possible with a mini-invasive DDA but needs to insert other pins in the femoral shaft to avoid any constraints on the femoral landmarks due to soft tissues.

The robotic arm optimizes the procedure. The robotic arm optimizes this procedure. A single reamer size corresponding to the pre-operative planning is used and will perform the acetabular reaming. The assisted robotic arm controls the surgeon’s hand regarding the orientation and progress of the reamer center of rotation during all reaming procedure.

The impaction of the final acetabular cup will also be carried out using the same control. The cup’s final orientation made by the pointer verifies the accuracy between the intraoperative values and the pre-operative templates with a threshold level of less than 2°. Insertion of the planned femoral stem is navigated in case of an enhanced procedure option. Trial acquisition could be performed during surgery in order to adjust the neck size concerning leg length and combined offset. Any re-adaptation of the planning is possible during the surgery if necessary. Finally, final implantation and control are performed. Landmarks are removed. The closure is performed.

### What is the current level of evidence of the benefit of robot-assisted surgery in the hip prosthesis?

Successful clinical outcomes following THA are usually multifactorial. Component positioning and insertion while respecting anatomical variations of the hip remain challenging. What is the real benefit in the use of robotic-assisted technology? Some factors must be analyzed such as:

- Accuracy and reproducibility of the implantation and complications.
- Bone economy.
- Management of combined anteversion.
- Learning curve.
- System adaptability regardless of the patient’s anatomy or morphology considerations.
- Benefits in the complication rate.
- Impact on functional outcome.
- Compatibility with ambulatory care and enhanced recovery after surgery.
- Cost-effectiveness evaluation.

Demographics of comparative studies between conventional and robotic-assisted THA are reported in Table 1.

#### Accuracy, reproducibility, and complications (Table 1)

In the early 90’s, the ROBODOC system was one of the first systems established for its use in joint arthroplasty. FDA approved its use in 2008 [39]. Compared to a hand-rasping group, some authors reported improved radiographic accuracy, implant fit, canal fill ratios, anteroposterior and axial stem alignment, vertical seating with this assistance [40]. A randomized study compared both the alignment and positioning of cementless THA using conventional manual THA versus the ROBODOC assisted THA [40]. This study revealed statistically significant improvement in positioning and fitting when the use of this robotic system was implemented. Also, Bargar et al. in a long-term study at a mean follow-up of 14 years reported the same conclusions and no failures for stem loosening and improvements in clinical outcomes in the robot group. Another advantage of this system is that this technology can operate using numerous prosthesis designs and manufacturers [41].

Despite the clinical and radiological benefits related to preoperative planning and accuracy of intraoperative procedure of this first generation of active and autonomous robotic assistance, this technology was discontinued. The high rate of complications have been described in other studies, such as muscle damage with higher dislocation rate (5.3%–18.0%) [42, 43], nerve damage (0.6%–7.0%) [42, 44], increased rates of infection and blood loss [42], heterotopic ossification [42], revisions (until 15%) [42], that were unlisted at the start of this experience [45] and reported up to 9% of patients by Banerjee et al. [2]. The revision rate was not increased [41]. This system made it challenging for orthopedic surgeons to intervene intra-operatively and modify any segment of the surgical plan. If some authors reported enhanced short-term outcomes in a randomized study with the insertion of short stem assisted by ROBODOC [46], recent independent studies are considered poor and the use of this type of system seems to be more restricted.

MAKO THA system (Stryker^®^, Mahwah, NJ) is a haptic/semi-active robotic-assisted system properly developed for both acetabular cup and femoral stem placement and advances in its technology have led to an improvement in preoperative planning and intraoperative techniques.

In a multicenter study involving 120 patients implanted with THA robotic assistance (MAKO^®^), cup positioning was compared between pre-operative planning, intraoperative assessment, and the postoperative 2D X-Ray measure [47].

The accuracy and reliability of the implantation of the cup were reported with a variation of only 3.5° in 95% of the cases (confidence interval 95%). The correlation between independent analysis of intraoperative data and scannographic measurements was recently reported in a prospective study [48].

Considering the Lewinnek safe zone, Illgen et al. [49] compared the placement of acetabular component with MAKO^TM^ robotic assistance (rTHA) and a conventional manual THA (mTHA). The rate of acetabular component placed within this zone was higher in the rTHA cohort (77%), followed by late mTHA (45%) and early mTHA (30%) (*P* < 0.001). No dislocations were reported at 2-year follow-up for rTHA compared to mTHA (3%–5%).

In a comparative study on THR placement conducted by Domb et al. [38] 1980 patients were implanted by six surgeons in a single unit. The influence of the technological assistance and the approach performed were analyzed. Six different surgical techniques were evaluated: conventional posterior prosthetic surgery, radioscopy-assisted posterior surgery, radioscopy-assisted anterior surgery (AAD), anterior navigated surgery (DAA), total anterior MAKO^TM^ robotic hip prosthesis (DAA), and total posterior robotic hip prosthesis. The rate of cup placements within the Callanan’s safe zone by both anterior and posterior approach was greater with the robotic assistance compared to all other modalities including navigation and fluoroscopy guidance. The adjustment of the hip length, location of center of rotation and combined offset were also analyzed reporting accuracy of the order of 1–0.7 mm, emphasizing the influence of positioning the hip center of rotation on the prosthetic stability.

Similarly, in a prospective matched cohort of 50 patients undergoing conventional manual THA and 25, a Mako^TM^ robotic-arm assisted THA, Kayani et al. [50] also reported improved accuracy placement of the cup according to the Lewinnek and Callanan safe zone. Recent studies reported similar results [51–56].

#### Bone economy

Bone saving is also considered as one of the goals of robotic assistance surgery. 3D planning enables the surgeon to adapt good reamer size which is considered as the key point considering:

- Adapted orientation of the native acetabulum.
- Restitution of the hip center of rotation.
- No oversize (choice of the realmer diameter at most 2 mm greater than the diameter of the femoral head)
- No anterior overhang of the cup.

Robotic assistance allows control of orientation and progression in reamer’s depth. The lack of control provides ovality of the reaming and protrusion of the reamer. Also, the linear respect of its progression produced by control of the reamers center of rotation; stave off any poor manipulation of the reamer by the surgeon. Following that, the stable implantation of the final cup is ensured.

Suarez-Aedo et al. [57] evaluated this bone preservation in a comparative study between a group of patients operating on using a conventional PTH or PTHC (*n* = 57) and a robotic assistance group or PTHR (*n* = 57). This study confirmed the value of measuring the diameter of the femoral head as a reference to the cup diameter. The study demonstrated that the MAKO^TM^ assisted THA allowed insertion of smaller acetabular cup sizes compared to that of the patient’s native femoral head size and thus greater preservation of bone stock.

#### Managing combined anteversion

Dislocation after primary THA is considered as the main complication with reported rates of 0%–5% in the literature. Debates around the validity of positioning safe zones to prevent instability are still ongoing and controversial [58, 59]. Restoration of joint biomechanics by appropriate implantation and soft-tissue preservation are the key points. Consideration of adapted combined anteversion including version of the stem appeared as an evolution in this research pattern [60].

Huge variability of native femoral rotations and anteversions is reported in the literature [61–63]. Determination of an ideal and patient-appropriate combined anteversion is challenging [63, 64]. Some elements such as lumbar sagittal balance and its progression over time, lumbo-pelvic complex stiffness, hip-spine mechanical relationship [20, 28] must be considered. However, the pre- and intraoperative control of this combined anteversion remains an attractive option.

Tsai et al. [24] comparing combined anteversion values with that of the contralateral hip after positioning with or without a robot-assisted system reported the difficulties and robotic assistance advantages in restoring the native hip geometry and.

Marcovigi et al. [65] studied the potential benefits of robot-assisted technology such as MAKO^TM^ for this control. In the case of cemented primary stem insertion, the influence of the native femoral version on the final stem version and on combined anteversion value was evaluated. Because of the reported high variability of the native femoral anteversion with a range of femoral native version from −20 to 40°, differences between stem version and femoral native neck version was significant (ranges reported from near 34 to −52°). Considering combined anteversion as the efficient target, Nodzo et al. [48] reported similar values for combined anteversion. A significant correlation was found between intraoperative and postoperative CT measurements of the femoral anteversion. Its management also depends on the design of the stem especially in the case of the uncemented stem. In order to adjust during the surgery the stem neck version, anatomical uncemented stem requires modular necks or a range of implants with different axial plane versions [66]. In the case of uncemented straight stems, Domb et al. [67] investigated a study using MAKO^TM^ assistance to evaluate the capacity to control axial positioning of the stem in order to adjust adapted intraoperative combined anteversion. Robotic guidance was effective in correcting the native femoral version aiming towards reaching a target of 15° despite the surgical approach used. The cemented stem could probably be considered as a good option for easier stem version adjustment [68]. No study is yet reported on the ability of navigation processes integrated into the robotic system to allow this type of adjustment using a cemented stem.

#### Management of hip offset and hip center of rotation

Offset in THA directly related to the center of rotation of the hip (COR) [19] influence the abductor muscle function. Abnormalities of the postoperative offset values can be sometimes the cause of implant wear or impingement. The restoration of the COR is involved in the longevity of the hip replacement as well as in appropriate muscle function [6, 69]. Navigation does not allow efficient control of the acetabular cup offset [16, 38] in opposition to robotic assistance, which allows optimal control of the hip center.

In a study performed by Nawabi et al. [25] on 12 acetabular components in 6 cadaveric hips to study the accuracy of desired Hip length and the offset using robotic THA. They found that the desired offset COR and leg length were obtained more accurately when using the MAKO^TM^ assisted technique compared to the conventional manual THA.

Combined offset adjustment is closely related to the design and/or placement of the stem. Consequently, the contribution of the robot for this adjustment is relative compared to every kind of other procedure [38]. However, El Bitar et al. [56] have noticed satisfactory predictive hip length and global offset measurements compared to values analyzed postoperatively on plain radiographs.

#### What about the learning curve...

In a single-surgeon retrospective study involving 100 patients that underwent consecutive PTHR surgery, Bukowski et al. [70] analyzed the influence of the learning curve on outcomes. Three patient groups were analyzed: G1: 100 first patients with PTHC, G2: 100 patients with PTHC, and G3: 100 first patients with PTHR. No luxation was reported at 1 year in the G3 group, unlike the other two groups.

Redmond et al. [71] analyzed the positioning of the implants, the duration of surgery, and the rate of complications since the beginning of his study of robotic procedures. This continuous single-operator series also grouped three patient groups: group A with the first 35 patients, group B the next 35, and group C the last 35. Considering the accuracy of the implantation (percentage of “outliers” of the safe zone), and the operating duration, the author reported a rapid learning curve and a procedure completely acquired after the first 35 cases.

For Kayani et al. [72], the learning curve of 12 cases was noted for acetabular cup positioning by robotic assistance. No learning curve effect for accuracy in restoring native hip biomechanics or achieving planned acetabular cup positioning and orientation was reported.

However, there was no difference between the different groups considering the technical problems (1 case was secondary to poor fixation of the femoral landmark in group A, 1 case of bad impaction of the cup in group A) or complications. In a recent meta-analysis, Han et al. [53] reported a significantly increased operating time of robotic procedures compared to the conventional techniques. This learning curve has to integrate sometimes the adjustment of surgeon practice with the use of a new design of the implant. Robotic THA binds the surgeon to a restricted choice of implant manufactured by the company distributing the robotic system. The robotic system may not be compatible with the surgeon’s routine implant system. The gain in experience translates into a gain of operating time [73]. In a retrospective study of 100 patients operated by a single experienced surgeon, Kong et al. [52] reported a cut-off of 14 cases to be competent in robot-assisted THA using the MAKO^TM^ robot (Stryker^®^, Mahwah, USA).

## Robotic support and BMI

Positioning the cup in obese patients may be more challenging. Gupta et al. [74] analyzed the accuracy of acetabular cup inclination and version in obese patients in primary posterior PTHR performed in 105 patients. The groups were created to study the body mass indexes (BMI, kg/m^2^) of BMI: ≥30 (*n* = 59), BMI: 30–35 (*n* = 34), and BMI: ≤35 (*n* = 12).

No statistical differences were found between the different groups regarding acetabular inclination (*P* = 0.43) or version (*P* = 0.95). BMI is not considered a limiting factor for the use of robotic assistance. On the contrary, its use makes the accuracy of implant positioning identical to non-obese patients.

## Complication rates

Despite the evolution of total hip arthroplasty techniques and technologies, complication rates as well as morbidity and mortality still play a major role in arthroplasties. Multiple studies have compared the complication rates between robotic-assisted THA and conventional manual THA. The main representative studies are reported in Table 2. Complication rate and description are not always reported in the published studies. However, most studies revealed that intraoperative complications decreased in the robotic THA in comparison to the manual THA group [42, 46, 75]. Honl et al. [42] and Nakamura et al. [43] found that the dislocation rate was increased in the robotic group. Analysis of these increased complications has to include other factors such as past robotic system and extended approach with soft tissue damage. Few studies [39, 44, 54] reported some intraoperative problems that can cause either intraoperative abortion of the robotic assistance, or inaccurate collection of data [76], or malposition or instability of the femoral stem (ROBODOC) or acetabular cup (MAKO^TM^, ROBODOC^TM^). The significant technical development of the system in recent years is evident. Recently, thanks to the high technologic development, robotic assistance enhances reliability and accuracy in addition to further limiting the complication rate when compared to non-robotic techniques.

**Table 2:** Demographic characteristics, follow-up and complications of reported main studies on robotic-assisted THA studies.

	Cup inclination		Cup anteversion	
	Conventional	Robotic	Conventional	Robotic
Tsai et al. [24]	42.2 (±6.7)	35.4 (±4.4)	24.5 (±17.4)	22.8 (±5.1)
Domb et al. [83]	42.6 (±5.4)	40 (±3.2)	13.3 (±7)	16.7 (±3)
Kamara et al. [51]	41.5 (±37.5)	40.5 (±14)	23.6 (±56.3)	19.4 (±19.5)
Kong et al. [52]	40.35 (±6.57)	41.52 (±4.05)	16.91 (±5.48)	19.12 (±4.45)
Domb et al. [38]	41.72 (±5.27)	40.13 (±3.33)	21.83 (±6.09)	16.91 (±3.87)
El Bitar et al. [56]	40.33 (±3.33)	38.9 (±3.2)	16.9 (±3.0)	20.3 (±2.8)
Elson et al. [47]	40.0 (±1.2)	39.9 (±2.0)	18.7 (±3.1)	18.6 (±3.9)
Kanawade et al. [54]	39.4 (±3.4)	38.8 (±1.6)	19.1 (±4.2)	20.7 (±2.4)
Nodzo et al. [48]	40.12 (±3.0)	40.4 (±2.1)	23.0 (±2.4)	23.2 (±2.3)
Redmond et al. [55]	40.5 (±4.3)	39.3 (±2.5)	21.3 (±4.0)	20.6 (±2.4)

### What are the real functional benefits of robot-assisted PTH surgery in the short-, medium-, and long-term?

If robotic assistance THA appears as a reliable tool for the accuracy of implant placement, does robotic assistance bring any real value to the remote results of surgery?

Bukowski et al. [70] reported significantly higher Harris functional scores in robotic surgeries (92.1/−1.8 vs. 86.1/−16.2; *P* = 0.002) at 1-year follow-up. Perets et al. [77] on a minimum of 2-year follow-up in a cohort of 162 patients with PTHR also report satisfactory short-term results. The FJS-12 [78] (Forgotten Joint Score) was comparable when compared to those in the literature in conventional surgeries. Based on 6 studies with a sufficient level of evidence, Han et al. [53] reported no significant differences.

The same results were obtained in a study performed by Honl et al. [42] in which better functional scores were obtained in the robotic-assisted group.

In a randomized study performed by Lim et al. [46] comparing functional outcomes between robotic-assisted THA and conventional THA groups at a follow-up of 2 years, both the Harris hip scores and the Western Ontario and McMaster Universities Osteoarthritis (WOMAC) scores showed no significant differences between the two groups.

The follow-up time of all comparative studies is of short-term. Long-term studies will be required to determine any long-term clinical outcome differences.

## Robotic THA ERAS-type procedure (Enhanced Recovery After Surgery), outpatient surgery

Outpatient total hip arthroplasty (THA) remains a controversial and challenging topic. The evolution of surgical practice, which includes improved recovery after surgery is currently in order to optimize the patient care pathway. Heng et al. reported no significant difference in length of stay between robotic-assisted THA and conventional THA [73]. Combination with adequate patient selection, minimally invasive surgery with preservation of the soft tissues, pain management, early rehabilitation, and rigorous patient selection appears now as a suitable process for lower limb arthroplasty [79]. During the last decades ERAS type procedure has hugely modified the early recovery of the patient. Unbiased evaluation of the early outcomes is difficult to analyze especially to determine the real part of the eventual benefit of the robotic assistance in the patient recovery. If Robotics does not yet appear as a factor influencing this approach, combined with adequate information and ERAS practice, it could play a major role in the strategy for quality of care.

## Robotic assistance: pedagogic support and data collection. MPS support

Robotic assistance is considered an educational tool necessitating addition by the junior surgeon of different factors of reasoning concerning the proper insertion of a total hip arthroplasty with regards to the patient’s anatomy. This allows students to master the insertion of implants both in the preoperative templating and during surgery. The robot controls the fellow’s hand during the entire procedure.

This system provides a collection of data validated postoperatively in numerous studies cited above. The surgeon can evaluate his surgical practice and analyze many correlations between the different data and their possible impact on short-, med- and long-term outcomes. The presence of an MPS (Mako Products Specialist) for managing the MAKOplasty THA Total Hip Prosthesis application during surgery remains mandatory even after reaching the learning curve. The intraoperative adjustment of the pre-operative planning and the control of possible intraoperative technical support problems make his presence mandatory.

## Cost-effectiveness

In our routine orthopedic practice, the use of robotic technology may improve accuracy, complication rates and provide better functional clinical scores but the use of this modality requires it to be cost-effective as well as easily accessible to hospital institutions. The integration of robotic surgery into common practice is closely related to its cost-effectiveness, especially in the short-term. Using the Markov decision analysis that integrates total cost of care, Moschetti et al. [80] revealed that robotic-assisted surgery for PKA is more cost-effective than manual process when the number of cases exceeds 94 annually, failure rates are less than 1.2% at 2 years, and patient age is measured. The costs of a robotic system can be compensated by saving money on decreased hospital length of stay, and projected savings required in a costly revision [7, 22]. Attraction of this new technology has to be taken into account. Centers for arthroplasty that contain robotics may generate increased numbers of these surgeries than centers deprived of such a robot. Third-party payers should also increase the reimbursement to hospitals and surgeons that employ this technology if this technology proves to reduce complications and prevent costly revisions [22].

Current studies are based on increasing patient cohorts but with setbacks caused by a small number of independent studies. The medico-economic assessment of clinical benefits and its costs burden is still not appropriately assessed. Taking into consideration the existing economic and social circumstances, cost-effectiveness evaluation remains an inevitable necessity.

## Limitations

The trend of robotic surgery is starting to evolve gradually, its use in hip arthroplasty might improve the accuracy of position as well as precision but this technology also presents several limitations. The main limitation of use might be related to its cost-effectiveness as well as its engineer dependency but also, multiple anatomical hip morphologies can present a difficulty for the robot to assess it.

For example, in dysplastic hips, the robot cannot determine the proper native femoral center of rotation, which presents a limitation of the use of this technology.

In addition, dysmorphic hip joints can also present a limitation of the use of robots because this technology is unable to detect the exact hip morphology, and finally, this technology cannot interpret the patient’s lumbo-pelvic sagittal balance, which can directly affect the optimal positioning of the acetabular cup.

The sagittal alignment of the spine and its relation with the pelvic tilt influence the acetabular cup orientation thus, the adapted orientation of the implant appears to be challenging and the choice of implant positions strictly depend on the surgeon during the planning. The integration of the spinopelvic parameters in the software should be considered from the perspective of robotic assistance.

Finally, additional randomized studies will be needed to confirm the definite advantages of the use of this technology and confirm the short- and long-term clinical results obtained.

## Conclusion

The use of robotic-assisted THA presents clear and evident benefits related to accurate implant positioning while maintaining minimal bone resection in addition to controlling femoral anteversion, length and size of the stem neck while estimating the proper length of the limbs. This technology allows early improvement in functional results that seems to enhance the patient’s recovery. The definite advantage of the use of this technology is still controversial and presents several limitations regarding its standard use in all hip morphologies and pathologies in addition to its availability and easy accessibility in all institutions.

Nevertheless, the use of conventional THA proved a shorter surgical time and a flatter learning curve required in addition to a reduction in cost and fewer pre-operative imaging required.

This overview encompasses the potential advantages and disadvantages of the use of robotics in orthopedic surgery in a variety of subspecialties. Despite some limitations and controversies around this topic, this technology presents promising outcomes and results with potential use in routine clinical application. Further randomized clinical studies with greater long-term follow-up will be necessary to confirm its utility in routine total hip arthroplasty.

## Conflict of interest

The first author is a consultant for Stryker and Mako users. No research funding, institution sort of support, financial support, or personal payment or benefits for this article has been received.

All other authors have no conflict to declare in relation to this paper.
